# The San Antonio Fair

**Published:** 1905-10

**Authors:** 


THE SAN ANTONIO FAIR.—The dates as finally agreed upon for San Antonio International Fair this year are November 18 to 29 inclusive.

The changes in dates by the management was made because of the change of date of the Dallas Fair.

